# 688 Child Friendly Burn Education to Reduce Parent and Child Anxiety

**DOI:** 10.1093/jbcr/iraf019.317

**Published:** 2025-04-01

**Authors:** Jenna Trost, Creola Woolery

**Affiliations:** Riley Hospital for Children; Riley Hospital for Children

## Abstract

**Introduction:**

Pediatric burn patients experience numerous stressors during their time in the hospital and during outpatient visits. For many children, burn injuries may be their first experience with pain. At this ABA verified pediatric burn center, children who sustained a burn injury demonstrated adverse reactions to non-painful procedures during therapy services which impacted a therapist’s ability to provide quality care. Research was completed in case study format to investigate if parent and child anxiety levels prior to therapy intervention were impacted by use of a burn specific children’s book.

**Methods:**

A sample size of 8 (n=8) was utilized to collect initial data on parent and child anxiety levels prior to non-painful therapy intervention. Written surveys were administered to parents during outpatient appointments to determine parent and child anxiety levels prior to custom compression garment measurement. A children’s book, “Gary the Garment Giraffe” was used as the intervention in this case study. The book was written and illustrated by a physical therapist and an occupational therapist to describe and illustrate the garment measuring process in child friendly language. Post-measures on parent experience were also collected.

**Results:**

The results of the pre/post survey are as follows: 75% of parents reported their child being “a little nervous” for the appointment and 37.5% of parents being “a little nervous” for the upcoming appointment. One (1) parent reported being very nervous for the appointment, and one (1) parent reported that their child was very nervous for the appointment. 100% of parents reported that the children’s book was very helpful in explaining the process of garments, and 100% of parents reported that they would recommend the book to be used with other patients.

**Conclusions:**

The initial results of this case study indicate the importance of continued collection of data of parent and child anxiety levels pre, during, and post non-painful procedures and the impact of child-friendly burn education on both parent and child anxiety levels with non-painful procedures. A randomized control trial has been developed to address the above and is currently in progress.

**Applicability of Research to Practice:**

A parent or child experiencing anxiety surrounding a traumatic injury can negatively impact a patient’s compliance with health care and therapy both immediately after injury and in the future. For children with burn injuries, it is important to identify effective interventions that reduce anxiety and stress during health care procedures and therapy. Understanding an effective approach to impacting pediatric mental health post traumatic injuries allows for compassionate care planning.

**Funding for the Study:**

N/A

